# Comparison of antibiotic resistance and molecular characteristics of *Escherichia coli* isolated from patients with UTI, ASB, and uropathic bloodstream infection

**DOI:** 10.3389/fmed.2025.1678401

**Published:** 2025-12-09

**Authors:** Sipei Wang, Sheng Zhao, Tinghua Ye, Xingxing Lou, Xinling Pan

**Affiliations:** 1 Department of Clinical Laboratory, Wenzhou Medical University Affiliated Dongyang Hospital, Dongyang, Zhejiang, China; 2 Department of Biomedical Sciences Laboratory, Wenzhou Medical University Affiliated Dongyang Hospital, Dongyang, Zhejiang, China

**Keywords:** *E. coli*, bloodstream infection, urinary tract infection, asymptomatic bacteriuria, virulence factor, antibiotic resistance

## Abstract

**Background:**

*Escherichia coli (E. coli)* could cause asymptomatic bacteriuria (ASB), urinary tract infections (UTIs), and bloodstream infections (BSIs). However, the characteristics of antibiotic resistance patterns and molecular features of *E. coli* strains among these three patient groups have not been clarified.

**Methods:**

Three patient groups were prospectively and consecutively enrolled, including: the BSI-B group (UTI with concurrent bacteremia), the UTI-U group (UTI without bacteremia), and the ASB-U group (asymptomatic bacteriuria). All isolated strains were confirmed as *E. coli* by matrix-assisted laser desorption/ionization time-of-flight mass spectrometry. Antibiotic susceptibility testing was performed against 18 agents using VITEK 2 Compact system with AST-GN13 cards. Additionally, next-generation sequencing was employed to characterize multi-locus sequence typing, phylogenetic groups, serotypes, and virulence factors.

**Results:**

There were 50 cases for each group enrolled in this study. The UTI-U isolates demonstrated significantly higher resistance rates to aztreonam (28.00% vs. 8.00%), ceftazidime (20.00% vs. 4.00%), cefepime (16.00% vs. 2.00%), and gentamicin (30.00% vs. 12.00%) compared to the BSI-B group (*P* < 0.05). Phylogenetic group B2 and sequence type ST131 predominated in the BSI-B and UTI-U groups, whereas ST1193 was predominant in the ASB-U group. Virulence gene analysis revealed a higher prevalence of exotoxin (*hlyABCD* and *cnf1*) and adherence (*papBCDEFGHJK*) genes in both the BSI-B and UTI-U groups compared to ASB-U (*P* < 0.05). Additionally, the BSI-B group uniquely displayed a higher carriage of the nutritional/metabolic genes *iroBCDEN*.

**Conclusion:**

*E. coli* isolates from different clinical sources showed variations in antimicrobial resistance and molecular characteristics, which would be helpful for UTI patients’ management.

## 1 Introduction

Urinary tract infection (UTI) is one of the most prevalent bacterial infections worldwide, with a global incidence of 1.6% (1), and its incidence is higher in women (estimated at approximately 10%) (2). From 1990 to 2021, both the incidence and mortality rates associated with UTI have shown an increasing trend (3). Severe UTI can progress to bloodstream infection (BSI), with community-acquired UTI being the predominant contributor, reported to account for 30–35% of adult bacteremia cases (4).

Uropathogenic *Escherichia coli* (UPEC) is the most common pathogen of UTI (5), but the clinical outcomes varied as some cases were companied by BSI and some present with asymptomatic bacteriuria (ASB). Studies have shown that 30% of ASB patients present with UTI symptoms (6). The different clinical outcomes could be potentially explained by varied bacterial determinants, including virulence determinants and resistance phenotypes.

In recent years, ESBL-producing *E. coli* are increasingly being detected in urine and blood (7), which enhances the risk of UTI relapse (8) and BSI (9). It has been reported that bacteria isolated from different specimen show distinct drug resistance patterns (10), making it difficult to select appropriate antibiotics, especially for patients with multiple sites of infections. Therefore, it is imperative to separately investigate the characteristics of strains obtained from urine and blood.

Current research on *E. coli* in UTI patients has primarily focused on strains originating from either intestinal or urinary sources (11). Although some studies have compared the antimicrobial resistance profiles of BSI isolates with those from urinary sources (10), such comparisons are limited by the heterogeneity of BSI strains, which encompass primary infections from non-urinary origins. To date, there were limited data on strains obtained from three infection status associated with UTIs: ASB, symptomatic UTI, and urinary tract-derived BSI. In this study, we collected *E. coli* from UTI patients with different status and analyzed their drug sensitivity phenotypes and molecular characteristics to provide data that will improve the treatment of UTI and BSI.

## 2 Materials and methods

### 2.1 Patient enrollment and classification

Three groups of patients who were admitted to Dongyang People’s Hospital from January 2023 to June 2024 were consecutively enrolled, with 50 participants enrolled into each group based on the following criteria: patients diagnosed with UTI combined with BSI (blood culture was positive), were designated as the BSI-B group; patients diagnosed with UTI without BSI were assigned to the UTI-U group; those tested positive in the urine culture but not diagnosed with UTI were assigned to the ASB-U group. For the BSI-B group, patients with infections unrelated to the urinary system were excluded. Moreover, patients’ age and gender were collected. The UTI diagnostic criteria can be found in Supplementary File 1.

The sample size was estimated based on the prevalence rates of virulence genes from our previous study (12) using G*Power software with a one-sided alpha of 0.05 and 80% statistical power. Considering practical constraints including the availability of qualified clinical isolates meeting our strict inclusion criteria during the study period, we ultimately included 50 isolates per group in the final analysis.

### 2.2 Specimen collection, bacterial culture, and species identification

The strains for group BSI-B were extracted from blood, and UTI-U and ASB-U strains were isolated from urine. The specimen collection and transfer were performed in line with the established guidelines by the health industry in the People’s Republic of China, namely the WS/T640-2018 standard for specimen collection and transit in clinical microbiology (13). Briefly, 20–50 mL urine samples were collected using sterile containers, and 5–10 mL blood samples were obtained into a blood culture vial (bioMérieux, France). The specimens were cultured under ambient conditions and promptly transported to the laboratory within 2 h. The specimens were then cultured on Columbia blood agar and chocolate agar plates (Kangtai, Wenzhou) and then incubated at 3°C under a 5% CO_2_ atmosphere for 24–48 h. The species were identified using the matrix-assisted laser desorption/ionization time-of-flight mass spectrometry (MALDI-TOF MS) when visible colonies were formed on the medium plates. The *Escherichia coli* ATCC8739 was used as the quality control strain. All the strains were derived from different patients.

### 2.3 Drug sensitivity testing

The resistance phenotype against 18 antibiotics (amikacin, ampicillin, aztreonam, amoxicillin, ceftazidime, ceftriaxone, cefotetan, ertapenem, cefepime, gentamicin, imipenem, ciprofloxacin, levofloxacin, nitrofurantoin, sulbactam-ampicillin, sulfamethoxazole- trimethoprim, tobramycin, piperacillin-tazobactam) and extended spectrum beta- lactamase (ESBL) were evaluated using the AST- GN13 card in vitek2 compact. The ATCC25922 strain was utilized to perform quality control.

### 2.4 Next-generation sequencing and gene annotation

Genomic DNA was extracted using the Cetyltrimethyl Ammonium Bromide (CTAB) method with minor modifications, and the DNA concentration, quality and integrity were measured using a Qubit Fluorometer (Invitrogen, United States) and a NanoDrop Spectrophotometer (Thermo Scientific, United States). Sequencing libraries were generated using the TruSeq DNA Sample Preparation Kit (Illumina, United States) and the Template Prep Kit (Pacific Biosciences, United States). Next, genome sequencing was conducted at the Personal Biotechnology Company (Shanghai, China) on the Illumina Novaseq platform. Data assembly was carried out following the removal of adapter contamination and data filtration using the Adapter Removal (14) and SOAPec (15). The filtered reads were assembled by SPAdes (16) and A5-miseq (17) for the subsequent design of scaffolds and contigs. Finally, the genome sequence was obtained following rectification using the pilon software (18). Phylogenetic and multilocus sequence typing (MLST) analyses were performed using the ClermonTyping program (19) and the MLST program (20), respectively. The Virulence Factors of Pathogenic Bacteria (VFDB) database (21) and The Comprehensive Antibiotic Resistance (CARD) database (22) were employed to obtain the pathogenicity genes and antibiotic resistance genes, respectively.

### 2.5 Statistical analysis

All statistical analyses were conducted using SPSS 26.0. Count data were presented as numbers and percentages and were compared using the Chi-square or Fisher’s exact test, with Bonferroni-corrected pairwise comparisons. Continuous data were expressed as medians accompanied by quartiles and were analyzed by an independent sample *Kruskal-Wallis* test. *P* < 0.05 was considered significant.

### 2.6 Ethics approval

This study, which involved human participants, was approved by the Ethics Committee of Dongyang People’s Hospital (No. 2022-YX-290) and conducted in accordance with the Declaration of Helsinki. All participants provided written informed consent, and data were collected anonymously.

## 3 Results

### 3.1 The differences in age and gender among the three groups

There was no significant difference in ages between group BSI-B and group ASB-U (Table 1). Notably, group BSI-B [74 (60, 82)] and group ASB-U [69 (62, 78)] were older compared with group UTI-U [57 (35, 65)] (*P* < 0.001). Notably, these three groups showed similar gender distribution.

**TABLE 1:** Comparative analysis of age and sex distribution across the three groups.

Index	BSI-B (50)	UTI-U (50)	ASB-U (50)	*P-*value
Age	74 (60, 82)	57 (35, 65)	69 (62, 78)	
				0.442 (BSI vs. ASB)
				< 0.001(UTI vs. ASB)
				< 0.001(BSI vs. UTI)
Gender (male)	11 (22.00)	8 (16.00)	6 (12.00)	0.402

BSI-B, urinary tract-associated BSI; UTI-U, urinary tract infection only; ASB-U, asymptomatic bacteriuria.

### 3.2 The antimicrobial drug resistance of *E. coli* among the three groups

The resistance rates of aztreonam (28.00%) and ceftazidime (20.00%) in the UTI-U group were significantly higher than those in the BSI-B group (8.00 and 4.00%, respectively (Table 2) (*P* < 0.05). The resistance rate of cefepime in UTI-U (16.00%) was higher than that in BSI-B (2.00%) and ASB-U (2.00%) (*P* < 0.05). The resistance rate of tobramycin in ASB-U (34.69%) was higher than that in BSI-B (14.00%) (*P* < 0.05) (Table 2).

**TABLE 2:** Distribution of antimicrobial resistance in *E. coli* across the three groups.

Antimicrobial type	Antimicrobial drug	BSI-B (50)	UTI-U (50)	ASB-U (50)	*P*-value
	ESBL	11 (22.00)	19 (39.58)	15 (31.25)	0.169
Penicillins	Amoxicillin	39 (78.00)	34 (72.34)	39 (82.98)	0.463
	Ampicillin	40 (80.00)	36 (73.47)	40 (80.00)	0.666
Aztreonam	Aztreonam	4 (8.00)_a_	14 (28.00)_b_	6 (12.24)_ab_	0.017
Cephems	Cefotetan	0	0	0	/
	Ceftazidime	2 (4.00)_a_	10 (20.00)_b_	5 (10.20)_ab_	0.040
	Ceftriaxone	12 (24.00)	21 (42.86)	14 (30.43)	0.126
	Cefepime	1 (2.00)_a_	8 (16.00)_b_	1 (2.00)_a_	0.007*
Carbapenems	Ertapenem	0	0	0	/
	Imipenem	0	0	2 (4.081)	0.107*
β-lactam/β-lactamase inhibitor combinations	Sulbactam/ampicillin	19 (38.00)	23 (46.94)	23 (46.00)	0.614
	Piperacillin/tazobactam	0 (0.00)	1 (2.00)	1 (2.04)	0.599
Aminoglycosides	Amikacin	0 (0.00)	1 (2.04)	1 (2.04)	0.550*
	To bramycin	7 (14.00)_a_	14 (29.17)_ab_	17 (34.69)_b_	0.051^
	Gentamicin	6 (12.00)_a_	15 (30.00)_b_	16 (32.65)_b_	0.035
Quinolones	Ciprofloxacin	19 (38.00)	21 (43.75)	26 (53.06)	0.316
	Levofloxacin	16 (32.00)	19 (38.00)	21 (45.65)	0.388
Nitrofurantoin	Nitrofurantoin	0 (0.00)	1 (2.04)	0 (0.00)	0.662*
Folate pathway inhibitors	Sulfamethoxazole/ trimethoprim	18 (36.00)	18 (36.00)	16 (32.65)	0.922

χ^2^ test was used to compare between groups.

*Fisher’s exact test. ^ BSI-B vs. ASB-U, *P* = 0.016. ab The groups with the same subscript letter indicate no significant difference between compared groups. BSI-B, urinary tract-associated BSI; UTI-U, urinary tract infection only; ASB-U, asymptomatic bacteriuria; ESBL, extended spectrum beta- lactamase.

However, no differences were observed in the distributed genes responsible for resistance against aminoglycoside, quinolones, sulfonamide and β-lactam antibiotics between the any two groups (Table 3 and Supplementary File 2).

**TABLE 3:** Distribution of antimicrobial resistance genes in *E. coli* across the three groups.

Antibiotic agent	Gene	Positive strains no. (%)			*P*-value
		BSI-B	UTI-U	ASB-U	
Aminoglycoside	*aadA2*	2 (4)	3 (6)	1 (2)	0.59
	*aadA5*	16 (32)	14 (28)	14 (28)	0.879
	*apha1-1AB*	1 (2)	3 (6)	4 (8)	0.352
	*strA*	19 (38)	20 (40)	17 (34)	0.819
	*strB*	19 (38)	20 (40)	18 (36)	0.919
	*addA1*	4 (8)	1 (2)	5 (10)	0.193
Fluoroquinolone	*parC*	50 (100)	50 (100)	50 (100)	
	*parE*	50 (100)	50 (100)	50 (100)	
	*gyrA*	50 (100)	50 (100)	50 (100)	
	*qnr*	3 (6)	1 (2)	4 (8)	0.352
Sulfonamide	*folP*	48 (96)	50 (100)	49 (98)	0.245
	*sul1*	19 (38)	18 (36)	16 (32)	0.815
	*sul2*	20 (40)	20 (40)	19 (38)	0.972
	*sul3*	1 (2)	2 (4)	4 (8)	0.35
β-lactams	*blaCMY-118*	6 (12)	5 (10)	5 (10)	0.932
	*blaCMY-47*	42 (84)	44 (88)	45 (90)	0.656
	*blaCTX-M-27*	6 (12)	8 (16)	3 (6)	0.284
	*blaCTX-M-55*	4 (8)	6 (12)	5 (10)	0.801
	*blaTEM-1*β	30 (60)	26 (50)	25 (50)	0.488

BSI-B, urinary tract-associated BSI; UTI-U, urinary tract infection only; ASB-U, asymptomatic bacteriuria.

### 3.3 Phylogenetic group and MLST results

Phylogenetic group analysis demonstrated that the *E. coli* strains isolated from the three groups were predominantly group B2. Specifically, Group B2 strains accounted for 66.00% in the BSI-B group, 60.00% in the UTI-U group, 50.00% in the ASB-U group, followed by group B1, accounting for 12.00, 8.00, and 16.00%, respectively (Table 4).

**TABLE 4:** Distribution of phylogenetic groups in *E. coli* across the three groups.

Phylogenetic group	BSI-B (%)	UTI-U (%)	ASB-U (%)	Total (%)
A	2 (4.00)	5 (10.00)	8 (16.00)	15 (10.00)
B1	6 (12.00)	4 (8.00)	8 (16.00)	18 (12.00)
B2	33 (66.00)	30 (60.00)	25 (50.00)	88 (58.67)
D	7 (14.00)	9 (18.00)	7 (14.00)	23 (15.33)
E	0 (0.00)	0 (0.00)	1 (2.00)	1 (0.67)
F	2 (4.00)	2 (4.00)	1 (2.00)	5 (3.33)

BSI-B, urinary tract-associated BSI; UTI-U, urinary tract infection only; ASB-U, asymptomatic bacteriuria.

A total of 35 sequence types (ST) and two unassigned types were detected (Supplementary File 3). The top five composition of *E. coli* ST in BSI-B group was as follows (Table 5): ST131 (10), ST1193 (8), ST69 (6), ST95 (6), ST73 (5); in UTI-U group was as follows: ST131 (11), ST1193 (10), ST69 (5), ST73 (4), ST10 (3); in ASB-U group was as follows: ST1193 (16), ST131 (6), ST69 (5), ST58 (4), ST10 (3).

**TABLE 5:** Distribution of MLST sequence types in *E. coli* across the three groups.

Ranking order	BSI-B (n)	UTI-U (n)	ASB-U (n)
Top1	131 (10)	131 (11)	1193 (16)
Top2	1193 (8)	1193 (10)	131 (6)
Top3	69 (6)	69 (5)	69 (5)
Top4	95 (6)	73 (4)	58 (4)
Top5	73 (5)	10 (3)	10 (3)

BSI-B, urinary tract-associated BSI; UTI-U, urinary tract infection only; ASB-U, asymptomatic bacteriuria.

### 3.4 The distribution of virulence genes of *E. coli* among the three groups

A total of 91 UPEC-related VF genes were identified (Supplementary File 4). The detection rates of five VF genes of Exotoxin (*hlyA, hlyB, hlyC, hlyD*, and *ncf1*) and eight VF genes of Adherence (*papB, papC, papD, papF, papG, papH, papJ, papK*) in BSI-B and UTI-U were higher than that in the ASB-U group (*P* < 0.05) (Table 6). Moreover, the BSI-B group exhibited higher detection rates of the nutritional/metabolic virulence genes *iroBCDEN* than the UTI-U and ASB-U groups, with a statistically significant difference observed specifically for *iroB* (*P* = 0.044). However, the carriage rate of *fepE* in the BSI-B was lower compared with that in the UTI-U and ASB-U groups (*P* = 0.011) (Table 6).

**TABLE 6:** Distribution of virulence genes in *E. coli* across the three groups.

VF function	VF name	BSI-B (%)	UTI-U (%)	ASB-U (%)	*P*-value
Exotoxin	*hlyA*	11 (22.0)_a_	16 (32.0)_a_	3 (6.0)_b_	0.005
	*hlyB/C/D*	11 (22.0)_a_	17 (34.0)_a_	3 (6.0)_b_	0.002
	*cnf1*	9 (18.0)	15 (30.0)	3 (6.0)	0.008
Adherence	*papB*	19 (38.0)_a_	16 (32.0)_a_	2 (4.0)_b_	< 0.001
	*papC/D*	23 (46.0)_a_	20 (40.0)a	4 (8.0)_b_	< 0.001
	*papF*	24 (48.0)_a_	23 (46.0)a	9 (18.0)_b_	0.002
	*papG*	21 (42.0)_a_	16 (32.0)a	3 (6.0)_b_	< 0.001*
	*papH/J*	23 (46.0)_a_	19 (38.0)a	4 (8.0)_b_	< 0.001
	*papK*	23 (46.0)_a_	20 (40.0)a	4 (8.0)_b_	< 0.001
Nutritional/metabolic factor	*iroB*	21 (42.0)_a_	13 (26.0)ab	10 (20.0)_b_	0.044
	*iroC*	21 (42.0)	14 (28.0)	10 (20.0)	0.052
	*iroD/E/N*	21 (42.0)	14 (28.0)	11 (22.0)	0.084
	*fepE*	45 (90.0)_a_	50 (100.0)_b_	50 (100.0)_b_	0.011*

χ^2^ test was used to compare between groups.

*Fisher’s exact test. ab The groups with the same subscript letter indicate no significant difference between compared groups. BSI-B, urinary tract-associated BSI; UTI-U, urinary tract infection only; ASB-U, asymptomatic bacteriuria. Human Organ Color Code: Red indicates infection; Black indicates asymptomatic. ATM, aztreonam; CAZ, ceftazidime; FEP, cefepime; TOB, tobramycin; GEN, gentamicin. Blue Color Scale Blocks: The numerical value represents the drug resistance rate. Darker blue shades indicate higher resistance rates. Red Color Scale Blocks: The numerical value represents the virulence gene carriage rate. Darker blue shades indicate higher carriage rates.

## 4 Discussion

**FIGURE 1:**
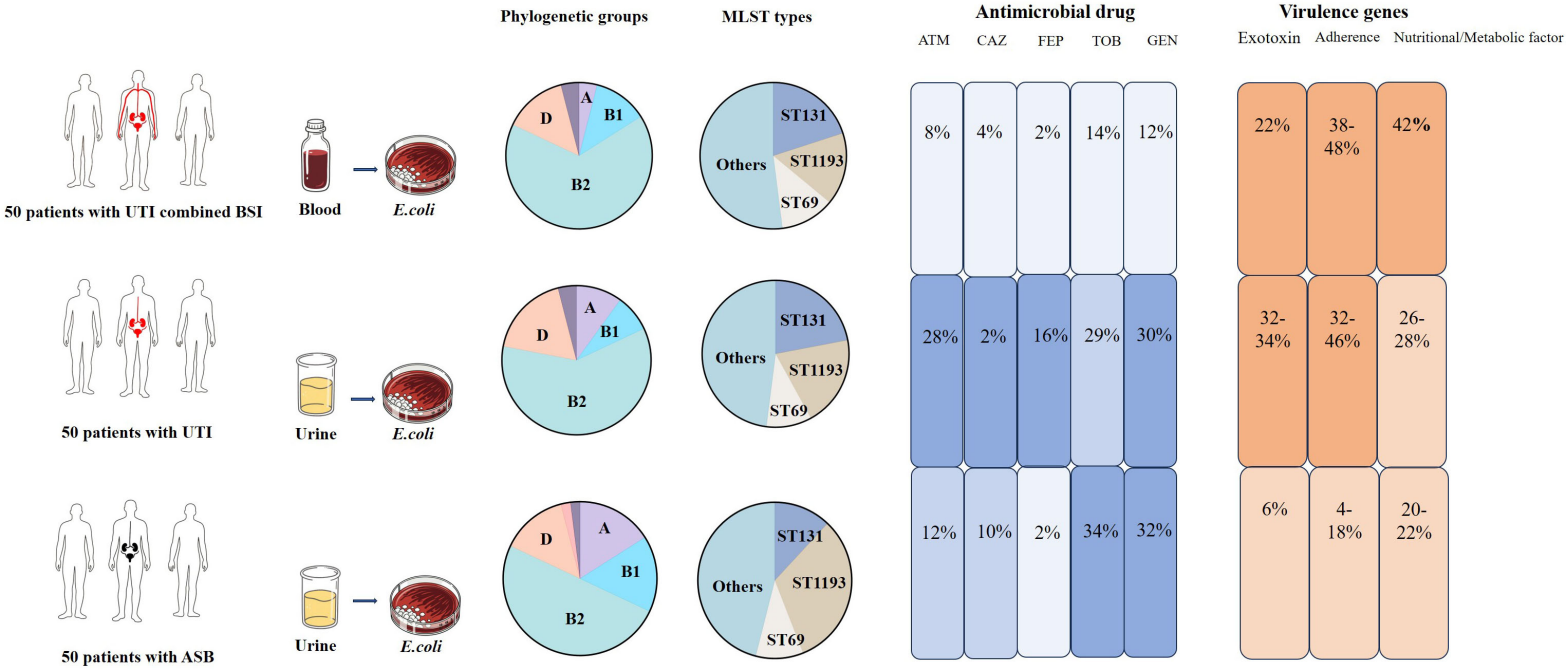
The summary findings in this study. Human Organ Color Code: Red indicates infection, black indicates asymptomatic. Antibiotic Abbreviations: ATM, aztreonam; CAZ, ceftazidime; FEP, cefepime; TOB, tobramycin; GEN, gentamicin. Blue Color Scale Blocks: The numerical value represents the drug resistance rate and darker blue shades indicate higher resistance rates. Orange Color Scale Blocks: The numerical value represents the virulence gene carriage rate and darker orange shades indicate higher carriage rates.

As summarized in the graphical abstract (Figure 1), three clinical statuses for UTI patients were included in this study, and our findings reveals distinct antimicrobial resistance and virulence gene profiles for *E. coli* strains from patients UTI, ASB, and BSI.

Both ASB and UTI were common in older adults, whereas ASB was uncommon in younger populations. Among healthy women, the prevalence of ASB increases with age, from under 1% in newborns to 10–20% in those aged 80 years (23). The finding that the median age of the BSI group was notably higher than that of the UTI group is consistent with the observations reported by Cheung et al. (24). This observation aligns with existing evidence that age is a significant risk factor for UTIs that are complicated by sepsis (25).

In this study, higher antimicrobial resistance rates were observed in UTI-derived strains compared to BSI isolates, which is contrary to a Finnish surveillance from 2008 to 2019 reported higher ESBL production rates in *E. coli* from blood cultures (1.6–8.6%) than from urine (1.0–7.2%) (7). Another study also found that levofloxacin resistance was higher in bloodstream isolates (10). This inconsistency could be explained by the different infection sites causing bloodstream infection. In detail, bloodstream isolates in those studies were not exclusively of urinary origin but included strains from biliary, intestinal, and other infections while all isolates in our study were obtained from patients with UTI.

The higher resistance observed in the UTI group may be attributable to its predominance of outpatients, who commonly receive empirical broad-spectrum antibiotic therapy such as fluoroquinolones and third-generation cephalosporins, thereby driving increased resistance (26). The widespread use of these antibiotics can contribute to the evolution of bacterial populations, leading to the development of subpopulations with diverse resistance phenotypes (23). Furthermore, poor adherence among outpatients often results in suboptimal infection management, which can further facilitate the rise of drug-resistant bacterial strains. Moreover, strains associated with bloodstream infections generally exhibit high virulence, as hypervirulent variants of bacteria often demonstrate lower levels of antimicrobial resistance. A previous study investigating the virulence-resistance relationship reported that pan-susceptibility to antibiotics was detected in 44.7% of high-virulence isolates compared to 57.7% of low-virulence isolates (25). In the present study, we found that more virulence genes were isolated from the BSI-B group compared with the UTI-U group. The observed lower resistance in the BSI group could be attributed to the relationship between bacterial virulence and drug resistance.

Based on the antimicrobial susceptibility profiles observed in the three groups and in alignment with the Chinese guidelines for UTI management (27), we recommend that antibiotic therapy be customized according to the infection site. In cases of upper UTIs, where there is a significant risk of concurrent BSI and confirmed urosepsis, it is crucial to initiate empirical antibiotic therapy without delay. Preferred initial regimens may include β-lactam antibiotics or a combination of β-lactams with β-lactamase inhibitors. For lower UTIs, which are characterized by higher resistance rates among bacterial isolates, we recommend obtaining urine cultures as soon as possible. This early step is essential for identifying the causative pathogen and enabling targeted antibiotic selection based on the susceptibility results. In the context of ASB, treatment is typically not advised according to existing literature, with the exception of pregnant women and patients scheduled for invasive urological procedures (28). In these specific situations, antibiotic therapy should also be informed by the susceptibility profile of the identified pathogen. *E. coli* strains from different phylogroups typically harbor distinct accessory gene pools (e.g., virulence factors, antibiotic resistance genes, metabolic genes), which contribute to the divergent antimicrobial resistance profiles and pathogenic potential.

Here, we found that the Phylogenetic group B2 was the dominant group in all three groups, which should be closely monitored as this group-caused infections have higher morbidity and mortality (29). Unfortunately, more than 70% of the isolates of complex UTI are the group B2 (30), which was similar to that causing BSI (53%) (27).

As the predominant ST in the BSI group in this study, ST131 is also prevalent among patients with *E. coli* caused bacteremia across multiple regions, including Shanghai (14/80) (31), Shanxi (15/76) (32), and Paris (exceeding 70%) (28). Moreover, some surveillance data indicate an increasing trend in the proportion of ST131 among bloodstream isolates over time (27). In addition to its high frequency in BSI, ST131 also exhibits a high prevalence in UTI, supported by our findings and data from other regions (33, 34). Nevertheless, the prevalent STs might vary from subgroups as ST1193 is the most common sequence type (25.83%) among UPEC isolates from female patients (35). Notably, all ST1193 strains demonstrated ciprofloxacin resistance in our study, consistent with its characterization as an emerging global fluoroquinolone-resistant clone. Therefore, quinolone antibiotics should be avoided in treating infections caused by ST1193 strains. Previous investigations indicated that ST1193 was the cause of community-acquired upper UTI in the elderly (36). But current guidelines do not endorse routine screening or prophylactic treatment for ASB (28). However, our findings reveal that the median age of the ASB cohort was over 60 years. Given the multidrug-resistant characteristics and increased pathogenicity of ST1193 strains in elderly populations, it is essential to closely monitor these individuals for the potential development of symptomatic UTIs.

UPEC strains harbor multiple virulence factors that contribute to pathogenesis. Our study found *hly, cnf1*, and *pap* genes to be more abundant in BSI-B and UTI-U groups than in the ASB-U group, consistent with previous reports (11, 37–39). This phenomenon reinforces their potential as key virulence determinants in symptomatic and invasive urinary tract infections. Furthermore, the *iroBCDEN* gene cluster, responsible for producing the high-affinity siderophore salmochelin, facilitates bacterial invasion of urothelial cells (40) and promotes systemic infection (41). Proteomics research suggests that iron uptake systems may contribute to the risk of UTI-related sepsis (42). In line with this, we observed an enrichment of the *iroBCDEN* genes in BSI-B isolates compared to UTI-U strains.

The distinct bacterial profiles observed across different UTI statuses carry distinct implications for clinical management. The higher abundance of adhesin-related virulence factors in UTI-U strains suggests a potential mechanism for enhanced urinary epithelial colonization, which may support the importance of ensuring adequate antibiotic courses to reduce recurrence risk in symptomatic UTI. In contrast, the generally low virulence of ASB-U strains aligns with current recommendations that many patients with ASB may not require treatment. Interestingly, some studies have proposed the potential use of such low-virulence strains as live biotherapeutic agents for recurrent UTI (43). For BSI-associated strains, the enrichment of siderophore genes such as those in the *iro* cluster suggests a potential role in systemic infection, which may warrant consideration of prompt antibiotic intervention in such cases. Future research integrating approaches with molecular docking and dynamics simulations, as demonstrated in related studies (44, 45), could further elucidate the mechanism underlying these observations and support the development of targeted agents.

However, this study has several limitations that should be acknowledged. First, the absence of clinical outcomes and longitudinal follow-up data prevented the assessment of associations between bacterial characteristics and patient prognosis. Second, the relatively small sample size, combined with the inability to perform one-to-one matching between blood and urine samples, further constrains the generalizability of our findings and underscores the need for validation in larger, well-structured cohorts. Finally, the single-center design carries an inherent risk of selection bias, which should be considered when interpreting the results.

## 5 Conclusion

In this single-center study, *E. coli* isolates from different clinical sources showed variations in antimicrobial resistance and molecular characteristics, which could be considered in the personalized management of UTI patients with different status.

## Data Availability

The original contributions presented in this study are included in this article/Supplementary material, further inquiries can be directed to the corresponding author.
