# Supplementary material for: Comparison of antibiotic resistance and molecular characteristics of Escherichia coli isolated from patients with UTI, ASB, and uropathic bloodstream infection
Source: Front Med (Lausanne). 2025 Dec 9;12:1678401. doi: 10.3389/fmed.2025.1678401 (PMC12722795; doi:10.3389/fmed.2025.1678401)
Supplement: Supplementary file 1 [file Table_1.docx]

The diagnosis of UTI was confirmed if patients had one of the following features: (1) the typical symptoms of UTI + pyuria (defined as >5 WBCs/HP) + positive urine nitrite test, (2) WBCs >10/HP in the urine sediment derived from the middle part of a cleaning centrifuge/typical symptoms of UTI + urinary bacterial count ≥10^5^/ml, (3) urinary bacterial count ≥10^5^/ml for two consecutive times, with similar bacterial subtypes in both counting , (4) the urine obtained from the bladder puncture tested positive in the culture, (5) presented with the typical symptoms of UTI + early morning clean midstream urine centrifuge urine sediment identifying the bacteria (bacteria >1/oil mirror field)(1). Patients with immunodeficiency conditions were excluded.

1. Wang H. Clincal Nephrorogy Pocket Book. Peking University Medical Press. 2010.
